# *﻿Malus* includes *Docynia* (Maleae, Rosaceae): evidence from phylogenomics and morphology

**DOI:** 10.3897/phytokeys.229.103888

**Published:** 2023-07-07

**Authors:** Guang-Ning Liu, Dai-Kun Ma, Chao Xu, Jian Huang, Bin-Jie Ge, Qiang Luo, Yu Wei, Bin-Bin Liu

**Affiliations:** 1 Beijing Botanical Garden, Xiangshan-Wofosi Road, Beijing 100093, China Beijing Botanical Garden Beijing China; 2 Beijing Floriculture Engineering Technology Research Centre, Beijing, China Beijing Floriculture Engineering Technology Research Centre Beijing China; 3 State Key Laboratory of Systematic and Evolutionary Botany, Institute of Botany, Chinese Academy of Sciences, No. 20. Nanxincun, Xiangshan Road, Beijing 100093, China State Key Laboratory of Systematic and Evolutionary Botany, Institute of Botany, Chinese Academy of Sciences Beijing China; 4 CAS Key Laboratory of Tropical Forest Ecology, Xishuangbanna Tropical Botanical Garden, Chinese Academy of Sciences, Xishuangbanna 666303, China CAS Key Laboratory of Tropical Forest Ecology, Xishuangbanna Tropical Botanical Garden, Chinese Academy of Sciences Xishuangbanna China; 5 Eastern China Conservation Center for Wild Endangered Plant Resources, Shanghai Chenshan Botanical Garden, No.3888 Chenhua Road, Songjiang District, Shanghai 201602, China Eastern China Conservation Center for Wild Endangered Plant Resources, Shanghai Chenshan Botanical Garden Shanghai China; 6 Institute of Agricultural Sciences, Xichang College, No. 1. Xuefu Road, Anning Town, Xichang City, 615013, China Xichang College Xichang China

**Keywords:** *Docynia*, *Malus*, nomenclatural transfer, phylogenomics, taxonomy

## Abstract

*Docynia* has been treated as a separate genus or merged into *Cydonia* or *Docyniopsis*. Our phylogenomic evidence from 797 single-copy nuclear genes and plastomes confirmed the sister relationship between *Docynia* and *Docyniopsis*. By integrating the phylogenomic and morphological evidence, we propose to accept a broad generic concept of *Malus* and merge *Docynia* into *Malus*. Three new combinations are also made here: *Malusdelavayi* (Franch.) B.B.Liu, *M.indica* (Wall.) B.B.Liu and *M.longiunguis* (Q.Luo & J.L.Liu) B.B.Liu.

## ﻿Introduction

**Figure 1.**
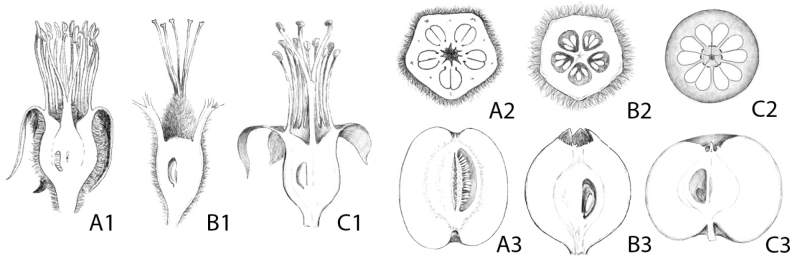
Morphological comparison amongst *Cydonia* (A1-A3), *Docynia* (B1-B3) and *Malus* (C1-C3) **A1, B1, C1** longitudinal section of carpel **A2, B2, C2** cross-section of fruit **A3, B3, C3** longitudinal section of fruit.

*Docynia* Decne. is a genus belonging to the apple subtribe Malinae and this genus is endemic to East and Southeast Asia (Yu and Ku 1974; Phipps et al. 1990; Gu and Spongberg 2003). Due to the easily distinguished multiple ovules per locule, 3-10 in *Docynia* (Fig. 1B2, B3) versus two in *Malus* Mill. (Fig. 1C2, C3), *Docynia* has been recognised as a separate genus in a series of taxonomic treatments (i.e. Decaisne (1874); Focke (1888); Koehne (1893); Rehder (1940, 1949); Yu and Ku (1974); Robertson et al. (1991); Kalkman (2004)). However, due to the multiple ovules per locule shared with *Cydonia* Mill. (Fig. 1A2, A3), Spach (1834) and Wenzig (1883) proposed an alternative taxonomic treatment, merging *Docynia* into *Cydonia*.

**Figure 2.**
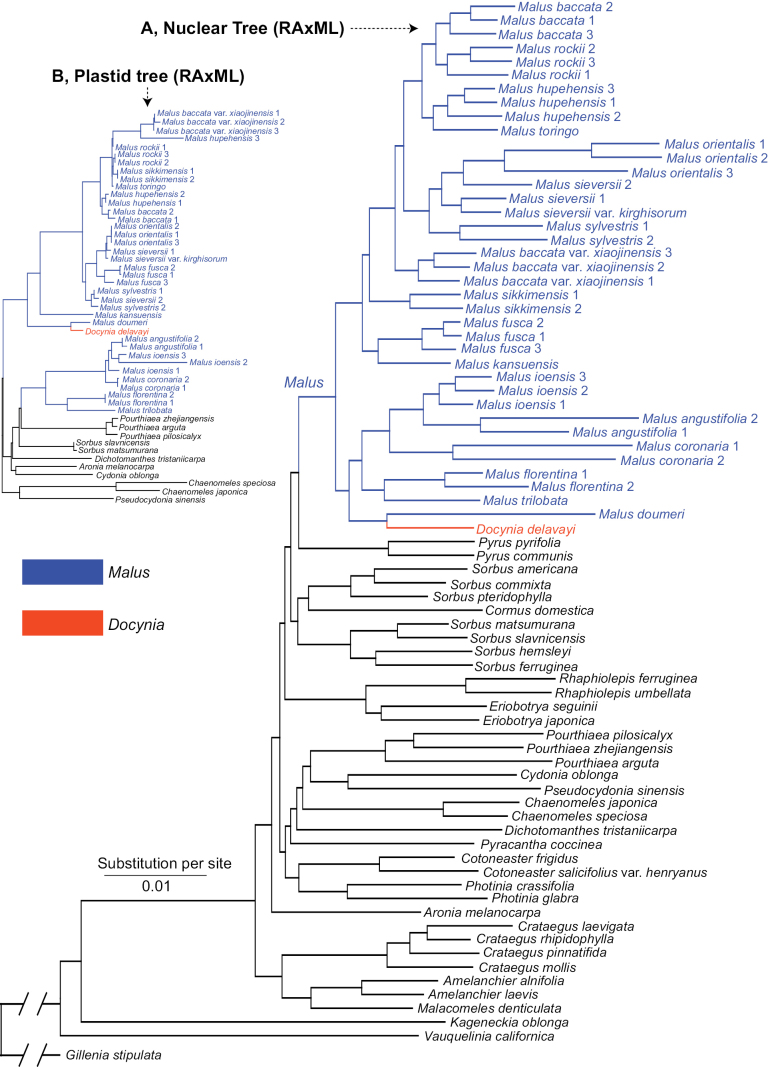
Maximum Likelihood (ML) tree of *Malus* within Maleae inferred from RAxML analysis using the concatenated 797 single-copy nuclear genes (SCNs) supermatrix (A), the upper left inset is a portion of the RAxML tree of *Malus*, based on the 78 concatenated plastid coding sequences (CDSs) supermatrix. (Adapted from figs 2 & 5 in Liu et al. (2022)).

Recent phylogenetic and phylogenomic studies presented strong topological discordance amongst nuclear/plastid genes and showed cytonuclear conflicts (referring to fig. 1 in Liu et al. (2022)). *Docynia* is closely related to *Docyniopsis* (C.K.Schneid.) Koidz. (= Malussect.Docyniopsis C.K.Schneid.), based on the plastomes and the nuclear sequences (Lo and Donoghue 2012; Liu et al. 2019, 2020a, 2020b, 2022; Jin et al. 2023). Several shared morphological characteristics have also supported their close relationship, i.e. cone-shaped non-adnate part of the ovaries (Fig. 1B1, C1), fully connate carpels (Fig. 1B1, C1), incurved and persistent calyx, numerous scattered sclereids throughout the flesh, juvenile leaves deeply lobed and similar flavonoid chemistry (Williams 1982; Robertson et al. 1991; Kalkman 2004). However, Jin (2014) proposed an alternative phylogenetic inference, based on the whole plastome, the sister relationship between *Docynia* and *Cydonia*. Additionally, Xiang et al. (2017) inferred a close relationship between *Docynia* and *Eriolobus* M.Roem., based on the transcriptomic data and this result provided another line of evidence for Schneider’s (1906) taxonomic transfer. However, Xiang et al. (2017) sampled only four apple-related species, *Malusbaccata* (L.) Borkh., *M.domestica* (Suckow) Borkh., *Docyniadelavayi* (Franch.) C.K.Schneid. and *Eriolobustrilobatus* M.Roem., the inferred phylogenomic topology based on this limited taxon sampling; thus, an accurate species relationship was not presented. Therefore, the argument that *Docynia* should be transferred to the genus *Eriolobus*, based on a strongly-supported sister relationship between the two taxa is untenable, as this evidence with limited taxon sampling is insufficient to justify a taxonomic reclassification proposed by Schneider (1906). Liu et al. (2022) sampled 39 individuals representing 18 wild species and provided a robust backbone of the apple and its allies in the framework of the tribe Maleae integrating 797 single-copy nuclear genes (SCN genes) and whole plastome data (Fig. 2). This phylogenomic analyses resolved the phylogenetic position of *Docynia*, placing it within *Malus* sensu lato (Liu et al. 2022).

In this study, we aim to transfer three currently-recognised species of *Docynia* to *Malus*.

## ﻿Materials and methods

We sampled 77 individuals in the framework of Maleae, of which 39 were apple-related species and the other 38 were outgroup species. All these 77 samples were performed for deep genome skimming (DGS) sequencing with 5-10G data for each sample. We assembled the whole plastome using NOVOPlasty v. 4.3.1 (Dierckxsens et al. 2016) and a successive assembly approach (Liu et al. 2021). Given the rich genomic resources in various lineages of Rosaceae, we screened 797 nuclear SCN genes from six genomes, *Malusbaccata*, *M.domestica*, *Pyrusbetulifolia* Bunge, *P.bretschneideri* Rehder, *P.ussuriensis* Maxim. × *P.communis* L. and *P.pyrifolia* (Burm.f.) Nakai. We assembled these 797 nuclear SCN genes for these 77 samples using HybPiper pipeline v. 1.3.1 (Johnson et al. 2016). The assembled sequences were then cleaned with a series of procedures, such as trimAL v. 1.2 (Capella-Gutiérrez et al. 2009), AMAS v. 1.0 (Borowiec 2016), TreeShrink v. 1.3.9 (Mai and Mirarab 2018) and Spruceup (Borowiec 2019). We combined the concatenated and coalescent-based methods for accurate phylogenetic inference. As for the concatenated-based method, we performed Maximum Likelihood (ML) tree inference using RAxML 8.2.12 (Stamatakis 2014) and IQ-TREE2 v. 2.1.3 (Minh et al. 2020). The shrunken trees from TreeShrink (Mai and Mirarab 2018) were used as input to estimate a coalescent-based species tree with ASTRAL-III (Zhang et al. 2018). The detailed parameters refer to the materials and methods in Liu et al. (2022).

## ﻿Results and discussion

**Figure 3.**
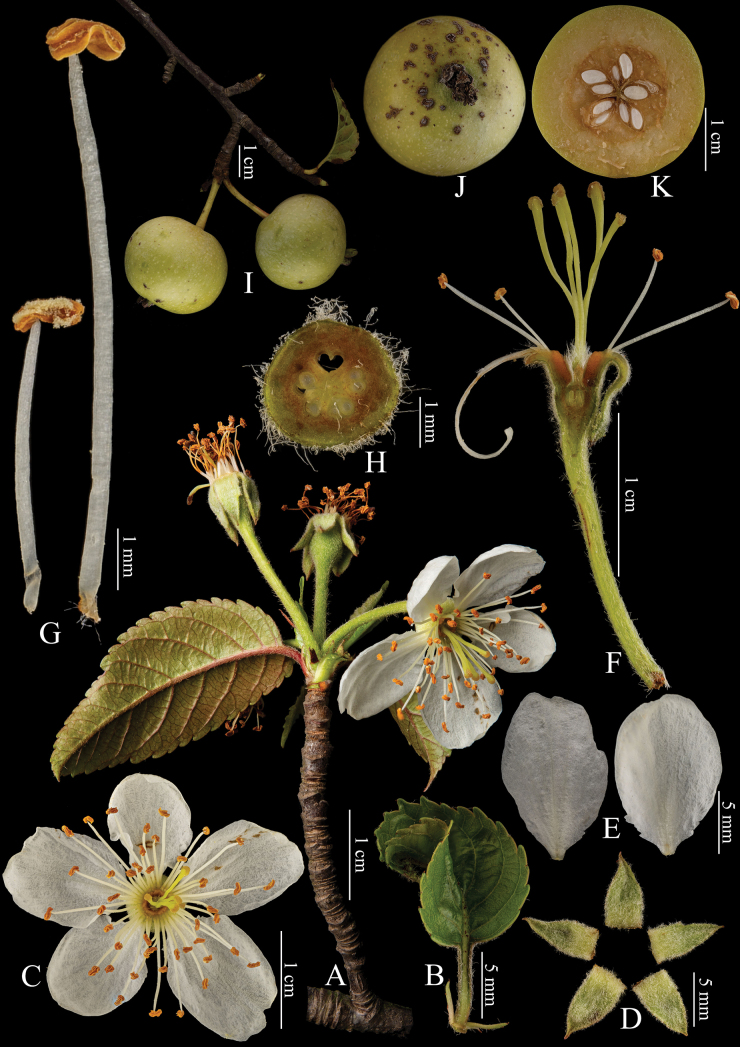
Structural comparison of the represented species in Malussect.Docyniopsis, *M.doumeri***A** inflorescence branch with young fruits **B** undeveloped leaves **C** flower **D** sepals (five) **E** petals **F** longitudinal section of flower **G** filament **H, K** cross-section of fruit in different stages **I** infructescence branch **J** the projected pome at apex and the persistent sepals. All photos credit to Bin-Jie Ge.

The phylogenetic relationship between *Docynia* and *Malus* has been controversial for two centuries. Our results revealed that all these nine nuclear and plastid trees in our study (Liu et al. 2022) demonstrated the paraphyly of *Malus* s.s., with *Docynia* nested within it (Fig. 2) and this was also confirmed in several recent molecular studies (Lo and Donoghue 2012; Xiang et al. 2017; Liu et al. 2020a). The sister relationship between *Docynia* and *Malusdoumeri* A.Chev. (= *Docyniopsis*) was confirmed either in the nuclear or plastid trees, except for the conflicting phylogenetic placement of the Docynia-Docyniopsis clade. As indicated in our previous study (Liu et al. 2022), the close relationship between *Docynia* and *Docyniopsis* (Fig. 3) was also supported by the morphological evidence, such as cone-shaped non-adnate part of the ovaries (Figs 1B1, C1, 3I), fully connate carpels (Figs 1B1, C1, 3K), incurved and persistent calyx (Fig. 3A, F, I, J), numerous scattered sclereids throughout the flesh, juvenile leaves deeply lobed and similar flavonoid chemistry (Williams 1982; Robertson et al. 1991; Kalkman 2004).

Despite the monophyly of narrowly-circumscribed small genera in *Malus* s.l., including *Chloromeles* (Decne.) Decne., *Docynia*, *Docyniopsis*, *Eriolobus* M.Roem. and *Malus* sensu stricto, we believe that such narrow generic concepts may be impractical for use by botanists, ecologists, conservation biologists and horticulturalists. Given the prevalence of reticulations in angiosperms, we recommend integrating multiple lines of evidence for accurate taxonomic treatments, including morphology, phylogenomics, cytology, biogeography and ecology, as proposed by integrative systematics (Wen et al. 2017). Traditionally, taxonomic circumscription was often focused solely on the taxonomic community, with little consideration given to its broader implications. However, today there is a growing recognition that taxonomic circumscription can have far-reaching effects on many aspects of biology, including conservation, ecology and evolution. By considering the needs of the broader biological community, taxonomic circumscription can help to ensure that taxonomic classifications are more valuable and relevant to a wider range of researchers and practitioners. An excessive inclination towards separating genera can hinder the advancement of research programmes for understanding evolution across all descendants stemming from a common ancestor. Additionally, by educating the general public about the importance of taxonomic circumscription, we can help foster a greater appreciation for biodiversity and its role in understanding and conserving it. In summary, taxonomic circumscription today should be viewed as a tool for serving the needs of both the taxonomic community and the broader biological community, as well as educating the general public about the importance of biodiversity and taxonomy (Wen et al. 2015, 2017; Funk 2018).

With all these considerations, we propose using the broad generic concept of *Malus*, which includes all members of *Malus* sensu Gu and Spongberg (2003) and the species in *Docynia*. We here formally transferred the three currently-recognised species of *Docynia* to *Malus* in the following text.

### ﻿Taxonomic treatment

#### ﻿ Malus indica

Taxon classificationPlantaeRosalesRosaceae

(Wall.) B.B.Liu comb. nov.

F3170CBA-8650-5A85-BC54-F100C67DC33A

urn:lsid:ipni.org:names:77322788-1

**Figure 4.**
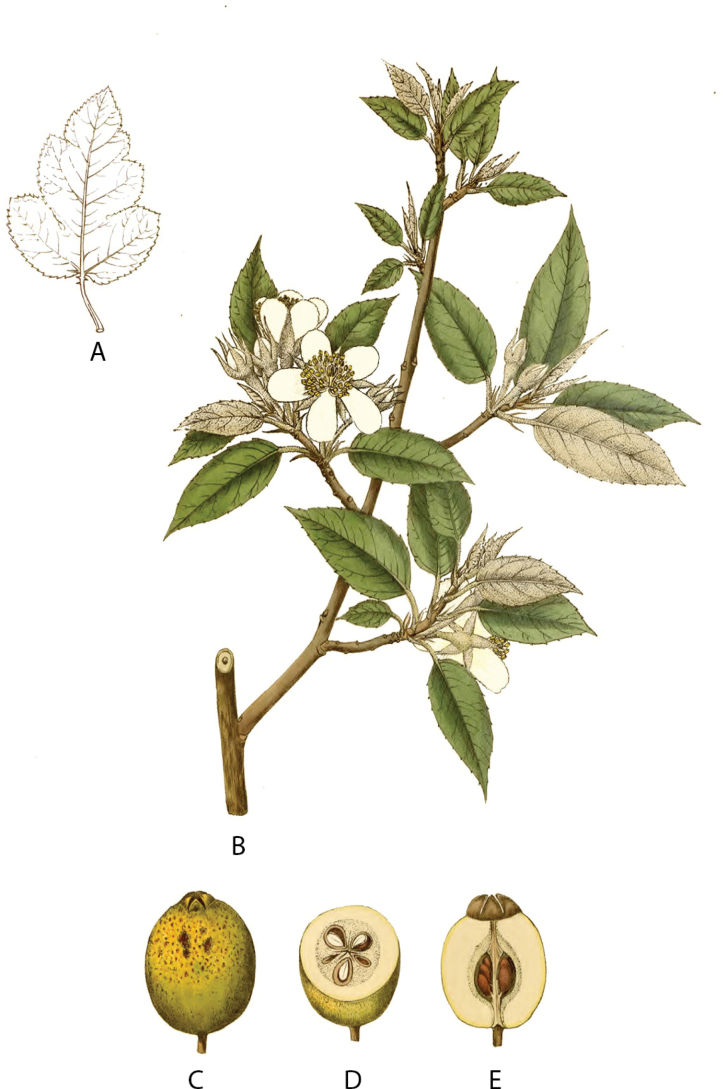
Holotype of *Malusindica* (redrawn from the illustration of Pl. Asiat. Rar. (Wallich 1831). 2: t. 173, 1831) **A** lobed-leaf **B** inflorescence branch **C** fruit **D** cross-section of fruit **E** longitudinal section of fruit.

**Figure 5.**
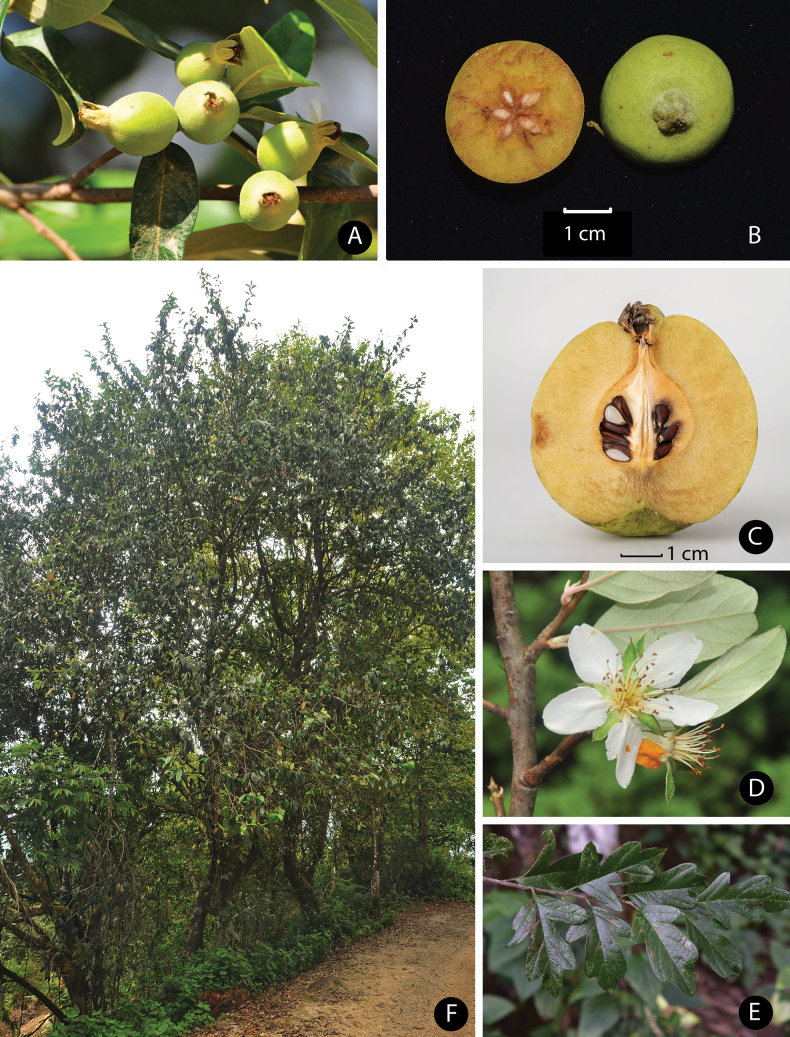
*Malusindica* (Wall.) B.B.Liu **A** young fruit **B** fruit (cross-section) **C** fruit (longitudinal section) **D** flower **E** leaf branch **F** overview of tree. Photo credits: **A, B, D, E** and **F** to Jian Huang; **C** to Bin-Jie Ge.

Figs 4 , 5 Chinese name: 多依; pinyin (spelled as it sounds): duo yi

≡ Pyrusindica Wall., Pl. Asiat. Rar. (Wallich) 2(8): 56 (1831). Type: Tab. 173 (holotype, Fig. 4). INDIA. “Khasia reg. temp. alt. 6000 pds”, *J.D. Hooker & T. Thomson 510* (**epitype, designated here**: M [barcode M0213698]!). Note 1. Image of the epitype available from https://plants.jstor.org/stable/10.5555/al.ap.specimen.m0213698.
≡ Cydoniaindica (Wall.) Spach, Hist. Nat. Vég. (Spach) 2: 158 (1834). Type: Based on Pyrusindica Wall.
≡ Docyniaindica (Wall.) Decne., Nouv. Arch. Mus. Hist. Nat. 10: 131 (1874). Type: Based on Pyrusindica Wall.
≡ Eriolobusindica (Wall.) C.K.Schneid., Ill. Handb. Laubholzk. 1: 728 (1906). Type: Based on Pyrusindica Wall.
= Docyniagriffithiana Decne., Nouv. Arch. Mus. Par. 10: 131 (1874). Type: INDIA. “Himalaya oriental.”, *Griffith 2082* (holotype: P [barcode P01819345]!; isotypes: E [barcode E00010836]!, K, CAL [accession no. 153563]). Image of the holotype available from https://plants.jstor.org/stable/10.5555/al.ap.specimen.p01819345.
= Docyniaindicavar.griffithiana (Decne.) Ghora, Bull. Bot. Surv. India 47(1–4): 150 (2005). Type: Based on Docyniagriffithiana Decne.
= Docyniahookeriana Decne., Nouv. Arch. Mus. Par. 10: 131 (1874). Type: INDIA. “Khasia, regio temp. alt. 5000 pds.”, *J.D. Hooker & T. Thomson 511* (holotype: P [barcode P01819346]!). Image of the holotype available from https://plants.jstor.org/stable/10.5555/al.ap.specimen.p01819346.
= Pyrusrufifolia H.Lév., Bull. Géogr. Bot. 25: 46 (1915), [Pirus]. Type: CHINA. Yunnan: “flane des coteaux arides à Lou-Pou, 3050 m, Juin 1912”, *E.E. Maire s.n.* (holotype: E [barcode E00010835]!). Image of the holotype available from https://plants.jstor.org/stable/10.5555/al.ap.specimen.e00010835.
= Docyniarufifolia (H.Lév.) Rehder, J. Arnold Arbor. 13: 310 (1932). Type: Based on Pyrusrufifolia H.Lév.
= Malusdocynioides C.K.Schneid., Bot. Gaz. 63: 400 (1917). Type: CHINA. Sichuan: “Szechuan australis: inter Kua-pie et Ta-tiao-ko, alt. ca. 2700 m, 23 Maji 1914”, *C.K. Schneider 1349* (holotype: K [barcode K000758093]!; isotype: A [barcode 00026465]!). Image of the holotype available from https://plants.jstor.org/stable/10.5555/al.ap.specimen.k000758093.
= Docyniadocynioides (C.K.Schneid.) Rehder, J. Arnold Arbor. 2(1): 58 (1920). Type: Based on Malusdocynioides C.K.Schneid.

##### Distribution.

Bhutan, China (Sichuan and Yunnan), India, Myanmar, Nepal, Pakistan, Sikkim, Thailand and Vietnam.

##### Note 1.

In the protologue of *Pyrusindica*, Wallich (1831) did not designate a specimen as the holotype, but only provided an illustration, which is considered to be the holotype (Fig. 4). However, the accurate identification of this species will be significantly impeded due to the limited morphological details in the illustration compared to the specimens (Turland et al. 2018). Consequently, it becomes necessary to select a single specimen as the epitype in order to distinguish it from its closest relatives, such as *Malusdelavayi* and *M.longiunguis*. Decaisne (1874) cited two specimens (*J.D. Hooker & T. Thomson 509* and *J.D. Hooker & T. Thomson 510*) while transferring this species to *Docynia* as *Docyniaindica*. Therefore, herein, we select a well-preserved specimen in the herbarium M (*J.D. Hooker & T. Thomson 510*: M0213698) as the epitype.

#### ﻿ Malus delavayi

Taxon classificationPlantaeRosalesRosaceae

(Franch.) B.B.Liu comb. nov.

D3E6397C-6E26-58A5-8BC0-C84B71D8572B

urn:lsid:ipni.org:names:77322789-1

**Figure 6.**
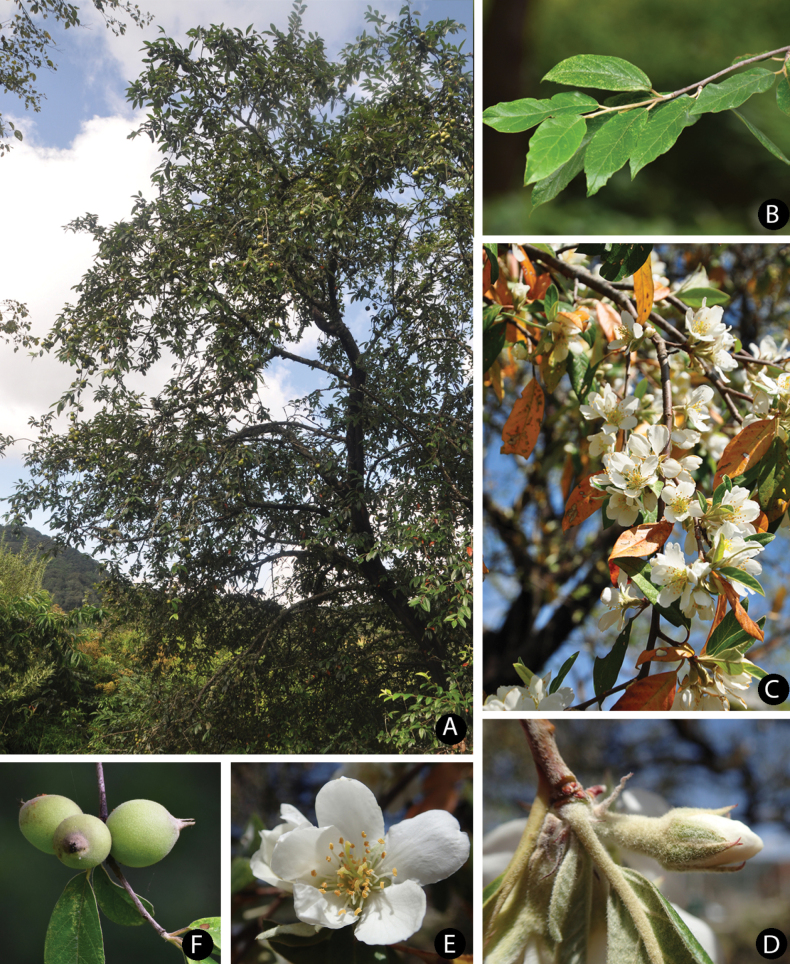
*Malusdelavayi* (Franch.) B.B.Liu **A** overview of tree **B** leaf branch **C** inflorescence branch **D** flower buds **E** flower **F** young fruits. Photo credits to Jian Huang.

Fig. 6 Chinese name: 云南多依; pinyin (spelled as it sounds): yun nan duo yi

≡ Pyrusdelavayi Franch., Pl. Delavay.: 227, t. 47 (1890), [Pirus]. Type: CHINA. Yunnan: “in montibus calcareis ad Mao-kou-tchang, supra Tapin-tze, prope Tali, alt. 2200 m.”, 14 April 1884, *P.J.M. Delavay 466* (**lectotype, designated here**: P [barcode P01819347]!; isolectotype: L [barcode L0019412]!); ibidem, *P.J.M. Delavay 890* (syntype: K [barcode K000758091]!); in silvis ad orientem versus montis Hee-chan-tong, alt. 2500 m, 5 April 1887 (syntypes: K [barcode K000758090]!, K [barcode K000758092]!). Image of the lectotype available from https://plants.jstor.org/stable/10.5555/al.ap.specimen.p01819347.
≡ Eriolobusdelavayi (Franch.) C.K.Schneid., Ill. Handb. Laubholzk. 1: 727 (1906). Type: Based on Pyrusdelavayi Franch.
≡ Docyniadelavayi (Franch.) C.K.Schneid., Repert. Spec. Nov. Regni Veg. 3: 180 (1906). Type: Based on Pyrusdelavayi Franch.
≡ Cydoniadelavayi (Franch.) Cardot, Bull. Mus. Natl. Hist. Nat. 24: 63 (1918). Type: Based on Pyrusdelavayi Franch.
= Cotoneasterbodinieri H.Lév., Bull. Géogr. Bot. 25: 44 (1915). Type: CHINA. Yunnan: “montagnes près de la frontière du Kouy-Tchéou; à Kiang-Ty”, 9 April 1897, *G. Bodinier s.n.* (holotype: E [barcode E00010834]!; isotype: A [barcode 00026464]!). Image of the holotype available from https://plants.jstor.org/stable/10.5555/al.ap.specimen.e00010834.

##### Distribution.

China (Guizhou, Sichuan, and Yunnan).

#### ﻿ Malus longiunguis

Taxon classificationPlantaeRosalesRosaceae

(Q.Luo & J.L.Liu) B.B.Liu comb. nov.

AB6148F9-73E0-5E9E-8105-D82DDB2036BB

urn:lsid:ipni.org:names:77322790-1

**Figure 7.**
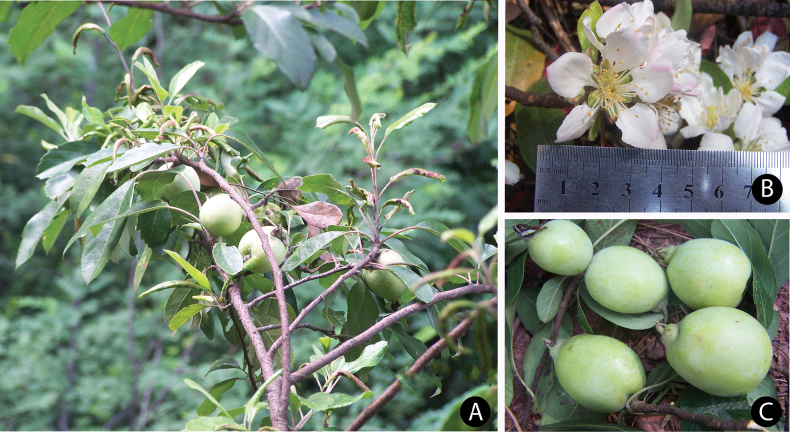
*Maluslongiunguis* (Q.Luo & J.L.Liu) B.B.Liu **A** fruit branch **B** flower **C** young fruit. Photos credit to Qiang Luo.

Fig. 7 Chinese name: 长爪多依; pinyin (spelled as it sounds): chang zhua duo yi

≡ Docynialongiunguis Q.Luo & J.L.Liu, Bull. Bot. Res., Harbin 31(4): 389 (2011). Type: CHINA. Sichuan: Xichang, Lushan, alt. 1860 m, 18 March 2010, *Q. Luo 010304* (holotype: PE [barcode 02362758]!). Note 2.

##### Distribution.

China (Sichuan).

##### Note 2.

In the protologue, the holotype is indicated as being deposited in the herbarium of Xichang College (HXCH, Luo et al. 2011); however, this holotype specimen was then sent to the China National Herbarium (PE).

## Supplementary Material

XML Treatment for Malus indica

XML Treatment for Malus delavayi

XML Treatment for Malus longiunguis

## ﻿Additional information

### Author ORCIDs

Guang-Ning Liu https://orcid.org/0009-0009-0765-0392

Dai-Kun Ma https://orcid.org/0009-0005-5523-508X

Chao Xu https://orcid.org/0000-0002-9678-4772

Jian Huang https://orcid.org/0000-0002-2253-4586

Bin-Jie Ge https://orcid.org/0000-0002-4232-3567

Qiang Luo https://orcid.org/0000-0001-5043-2539

Yu Wei https://orcid.org/0000-0002-2013-4829

Bin-Bin Liu https://orcid.org/0000-0002-0297-7531

### Data availability

All of the data that support the findings of this study are available in the main text or Supplementary Information.
